# Teacher Written Feedback on English as a Foreign Language Learners’ Writing: Examining Native and Nonnative English-Speaking Teachers’ Practices in Feedback Provision

**DOI:** 10.3389/fpsyg.2021.629921

**Published:** 2021-02-04

**Authors:** Xiaolong Cheng, Lawrence Jun Zhang

**Affiliations:** ^1^School of Foreign Languages, Hubei University of Technology, Wuhan, China; ^2^Xi’an Jiaotong University, Xi’an, China; ^3^Faculty of Education and Social Work, University of Auckland, Auckland, New Zealand

**Keywords:** native English-speaking teachers, non-native English-speaking teachers, teacher written feedback, teachers’ practices, writing in a foreign language

## Abstract

While previous studies have examined front-line teachers’ written feedback practices in second language (L2) writing classrooms, such studies tend to not take teachers’ language and sociocultural backgrounds into consideration, which may mediate their performance in written feedback provision. Therefore, much remains to be known about how L2 writing teachers with different first languages (L1) enact written feedback. To fill this gap, we designed an exploratory study to examine native English-speaking (NES) and non-native English-speaking (NNES) (i.e., Chinese L1) teachers’ written feedback practices in the Chinese tertiary context. Our study collected 80 English as a foreign language (EFL) students’ writing samples with teacher written feedback and analyzed them from three aspects: Feedback scope, feedback focus, and feedback strategy. The findings of our study revealed that the two groups of teachers shared similar practices regarding feedback scope and feedback strategies. Both NES and NNES EFL teachers used a comprehensive approach to feedback provision, although NNES teachers provided significantly more feedback points than their NES peers and they delivered their feedback directly and indirectly. However, their practices differed greatly with regard to feedback focus. Specifically, when responding to EFL students’ writing, NES teachers showed more concern with global issues (i.e., content and organization), whereas NNES teachers paid more attention to linguistic errors. With a surge in the recruitment of expatriate NES and local NNES English teachers in China and other EFL countries, our study is expected to make a contribution to a better understanding of the two groups of EFL teachers’ pedagogical practices in written feedback provision and generate important implications for their feedback provision.

## Introduction

Anchored in the educational process, feedback is an essential pedagogical practice in classroom-based instruction, which contributes to students’ learning and facilitates their achievement (Hattie and Timperley, 2007). Specifically, with the help of feedback, students can have a deeper insight into their weaknesses and strengths in learning, and how to improve their learning outcome (Yu et al., 2018). In the realm of second language (L2) writing, teacher written feedback, as a widely used intervention method, scaffolds L2 learners’ writing process and enhances their writing performance (Zhang, 2013, 2018; Hyland and Hyland, 2019). Thus, recent years have witnessed the proliferation of studies on teacher written feedback, particularly on written corrective feedback (WCF, i.e., feedback on linguistic errors) in L2 writing. In the extant literature, there is a back-and-forth debate over the WCF effectiveness, triggered by Truscott (1996). Having synthesized several early empirical studies, he vehemently repudiated the practice of WCF, arguing that WCF is ineffective and even harmful to L2 writing, so teachers should refrain from it. Currently, a consensus has been reached that WCF has a beneficial effect on text revision and new pieces of writing (e.g., Bitchener and Knoch, 2009a, b, 2010a, 2010b; Van Beuningen et al., 2012; Shintani and Aubrey, 2016; Bonilla López et al., 2018; Benson and DeKeyser, 2019; Li and Roshan, 2019; Karim and Nassaji, 2020).

To date, ample studies on teacher written feedback have adopted an intervention design to examine the relative efficacy of different types of WCF. However, relatively little attention has been paid to how practitioners implement written feedback in their specific teaching contexts (Lee, 2013, 2020; Alshahrani and Storch, 2014; Yu et al., 2020), and such limited studies tend to be conducted outside of mainland China (Mao and Crosthwaite, 2019; Yu et al., 2020). Given the powerful influence of context on teachers’ teaching practices (Borg, 2015; Bao, 2019; Sun and Zhang, 2019; Gao and Zhang, 2020), more relevant studies in the mainland Chinese EFL context are called for.

Furthermore, in the wake of globalization and internationalization, English has been established as an international lingua franca in the modern world (Rubdy et al., 2011; Schreier et al., 2020). Annually, EFL countries hire a large number of NES teachers to teach English and they have become an integral part of community of English teachers in these countries (Rao and Yuan, 2016; Zhang and Zhang, 2020). The sharp increase in the number of NES teachers initiates the innovations in EFL teaching and brings the comparison between the expatriate NES teachers’ and their local NNES peers’ teaching behaviors to the fore (Clark-Gareca and Gui, 2019; Zhang and Zhang, 2020). The available studies have shown that although NES and NNES teachers adopt different pedagogical practices in their instruction, the two groups of teachers complement each other as well as contribute their own advantages to language teaching (Shi, 2001; Zhang and Elder, 2011; Kung, 2015; Su, 2019; Zhang and Zhang, 2020). Unfortunately, while a growing number of NES teachers are recruited to teach EFL writing in China and other similar EFL countries due to their higher English proficiency and better understanding of English writing conventions and genres (Rao and Yuan, 2016; Zhang, 2016; Rao and Li, 2017), few studies have examined the similarities and differences between NES and NNES EFL writing teachers’ written feedback practices. Considering that teachers’ written feedback practices are conditioned and influenced by their sociocultural and language backgrounds (Lee, 2014, 2017; Hyland and Hyland, 2019), such an examination is warranted. It is believed that the findings of this study can shed light on a better understanding of how teachers’ unique sociocultural background impacts their feedback provision.

To bridge these gaps, we conducted an exploratory study to compare and understand how NES and NNES EFL teachers implemented written feedback in the mainland Chinese tertiary EFL writing classrooms. This study is of considerable practical value. At present, much emphasis has been placed on L2 writing as a construct of L2 learning by the academic and pedagogical communities (Zhang, 2013, 2016). The in-depth analysis of NES and NNES EFL teachers’ written feedback practices affords insights into facilitating their written feedback provision, which has been on the agenda of the educational reform on teaching EFL writing in China in recent years (Zhang, 2016).

## Literature Review

### Teacher Written Feedback

In the recent literature on teacher written feedback, researchers have managed to classify written feedback and identified several recurring themes: Feedback focus, feedback scope, and feedback strategy (e.g., Ellis, 2009; Sheen, 2011; Bitchener and Ferris, 2012; Lee, 2017; Mao and Crosthwaite, 2019). These three themes are also the foci of this study, which are explained in detail in the following paragraphs.

Researchers define feedback focus as what teachers focus on when they provide written feedback (Yu and Lee, 2014). Generally speaking, when assessing L2 learners’ writing or offering feedback, researchers or teachers look at the different dimensions of writing (i.e., language, content, and organization) (Jacobs et al., 1981; East, 2009). Moreover, they further categorize *language* as a local level focus; *content* and *organization* are regarded as attention to global issues of writing (Wallace and Hayes, 1991; Butler and Britt, 2011). Accordingly, there are two types of feedback with regard to focus: Local feedback (also termed WCF) and global feedback. In the existing literature, global feedback is under-researched compared with WCF (Li and Vuono, 2019). Of the limited studies on global feedback, several have investigated the relative effectiveness of global feedback and WCF and these studies have revealed that global feedback has weaker effects on improving high-order dimensions of writing in comparison with the effects of WCF on writing accuracy (e.g., Lee, 1997; Ashwell, 2000; Sampson, 2012). According to Geng’s (2017) and Zhang’s (2018) interpretations, such a finding may be associated with L2 learners’ perceptions of revision. Specifically, they tend to focus their attention on linguistic errors, so they probably have little attention to problems in high-order dimensions of writing. Such a flawed revision schema probably makes students underestimate and even ignore their global problems in writing. From our perspective, their interpretations are based on a well-established theoretical understanding represented in Trade-off Hypothesis proposed by Skehan (1998, 2009). According to this hypothesis, students have limited attentional resources. Thus, when paying much attention to linguistic errors in revision, they will probably have few attentional resources for global problems. Such a focus on revision may impair their performance in the global level of writing relating to the content and organization of their texts (see also Rahimi and Zhang, 2018, 2019).

Feedback scope refers to whether teachers should correct a wide range of errors (i.e., comprehensive/unfocused feedback) or only focus on a few errors and leave others uncorrected (i.e., selective/focused feedback). To date, a large number of scholars appear to prefer focused feedback and they have provided theoretical and empirical justifications for their preference. Theoretically, with focused feedback, L2 learners can probably avoid cognitive overload and they have additional cognitive resources to process new information (Sheen, 2007). Empirically, they have evidenced its effectiveness in improving accuracy in target linguistic structure(s) by many (quasi-) experimental studies (e.g., Bitchener and Knoch, 2009a, b, 2010b; Shintani and Aubrey, 2016; Benson and DeKeyser, 2019; Suzuki et al., 2019).

Despite this, some researchers have cast doubt on focused feedback because of its lack of ecological validity (e.g., Ferris, 2010; Storch, 2010; Hartshorn and Evans, 2015; Karim and Nassaji, 2020; Lee, 2020). Ecological validity refers to the extent to which results or conclusions obtained from an experiment or a quasi-experiment in a laboratory context can also be generalized to a naturally occurring situation in everyday life (Dörnyei, 2007; Creswell, 2014). In the field of teacher written feedback in L2 writing, the beneficial effects of focused feedback have been documented in many experimental or quasi-experimental studies (e.g., Sheen, 2007; Bitchener and Knoch, 2009a, b, 2010a, 2010b; Shintani and Aubrey, 2016; Suzuki et al., 2019). However, such an instructional practice targeting a limited number of errors does not correspond to the reality of L2 writing classrooms (Ferris, 2010). Specifically, in the real L2 writing classrooms, students tend to make various errors in their writing. Teachers assume the responsibility to help students “produce high-quality final writing products” (Bitchener and Ferris, 2012, p. 117) and the ultimate goal of teachers’ provision of written feedback is to improve their students’ overall writing performance rather than their performance in some specific linguistic structures (Storch, 2010; Van Beuningen, 2010). In this sense, it is not sufficient for teachers to adopt focused feedback on a small scope of errors in the L2 writing context. In stark contrast to the burgeoning studies on focused feedback, relatively few studies have examined the effects of comprehensive feedback, reporting that comprehensive feedback benefits L2 learners’ general writing accuracy in text revision and/or new pieces of writing (Van Beuningen et al., 2012; Frear and Chiu, 2015; Bonilla López et al., 2018; Karim and Nassaji, 2020).

In addition to the extent to which written feedback should be provided, teachers are faced with what strategies should be adopted to deliver written feedback. Broadly speaking, there are two types of feedback strategies: Direct and indirect feedback. The former refers to the provision of direct corrections to errors, while the latter means the identification and indication of errors without giving corrections (Lee, 2017). There are different ways to realize direct and indirect feedback strategies and they vary with the feedback focus. When addressing global problems directly, teachers can make overt corrections or provide feasible suggestions about how to solve them (Wang, 2015; Geng, 2017), while in terms of direct feedback on linguistic errors, it can be represented by different forms including presenting correct answers directly, deleting redundant or erroneous items, and inserting omitted items (Ellis, 2009; Sheen, 2011; Lee, 2017). In response to global issues indirectly, teachers just identify and indicate them without corrections or suggestions for improvement (Wang, 2015; Geng, 2017). As for indirect feedback on language, it can be formulated by highlighting the errors with/without metalinguistic clues or explanations (Sheen, 2011; Lee, 2017).

Currently, a firm conclusion regarding the relative effectiveness of direct and indirect feedback has not yet been drawn. Those who endorse indirect feedback claim that it prompts students to engage themselves in the process of error correction more profoundly than direct feedback, which contributes to their better understanding of the nature of the errors that they make (Lalande, 1982; Ferris, 1995). In contrast, advocates for direct feedback argue that direct feedback provides learners with input, enabling them to avoid misunderstanding and confusion when attending to feedback and internalize the correct forms immediately (Chandler, 2003; Bitchener and Knoch, 2010a).

### L2 Teachers’ Written Feedback Practices

As aforementioned, in comparison with studies on the effects of teacher written feedback, few studies have been conducted to observe how L2 writing teachers enact such a practice (Evans et al., 2010; Mao and Crosthwaite, 2019). In a case study to explore EFL writing teachers’ actual practices in regard to written feedback in a Saudi university, Alkhatib (2015) revealed that teachers showed more concern with linguistic errors than problems in content and organization when providing feedback. Furthermore, they provided feedback comprehensively, combining both direct and indirect feedback strategies. Similarly, conducted in the Hong Kong EFL context, Lee’s (2008, 2011) studies found that in feedback provision, secondary school teachers concentrated on local issues and provided feedback in a comprehensive way, in which direct feedback predominated. Different from these studies focusing on EFL teachers, Junqueira and Payant’s (2015) study drew upon students’ writing samples to examine how an ESL teacher enacted written feedback in her instructional context and it revealed that the teacher paid much more attention to local issues than global issues and mixed both direct and indirect feedback when responding to students’ writing.

Surprisingly, little research in this line has been carried out in mainland China, so we have little knowledge about teachers’ performance in relation to offering written feedback in this context. Here, one research deserves our attention and comment due to its particular relevance to our study. Mao and Crosthwaite’s (2019) study, to our knowledge, is the first one to address this issue in the mainland Chinese EFL writing classrooms. Their study employed a case study approach to investigate how five tertiary EFL teachers performed written feedback in their pedagogical settings. Collecting and analyzing data from 100 students’ writing samples (20 each teacher), the study showed that the teachers used a focused approach to provide feedback and the amount of indirect feedback that they offered was larger than that of direct feedback. In terms of feedback focus, the teachers gave more importance to local errors (i.e., grammar and vocabulary), in comparison with global issues. Such findings extend our knowledge about L2 writing teachers’ instructional practices regarding feedback provision in mainland China, a rapidly growing but under-explored EFL context. Despite their contributions, they fail to tell the whole story. To be specific, their study only focused on and examined NNES (Chinese L1) EFL teachers’ actual written feedback practices but did not take their NES counterparts’ practices into consideration.

### NES and NNES Teachers’ Assessment in L2 Writing

Research into assessment can enable insights into how teachers may approach giving feedback. In this section, we review studies comparing NES and NNES teachers’ assessment practices in L2 writing. Language assessment has been regarded as a social activity, in which teachers’ sociocultural and linguistic backgrounds play an important role in their assessment practices (McNamara, 2001; Su, 2019). In the filed of L2 writing, many studies have examined how teachers’ language backgrounds affected their scoring of English writing, but they have yielded mixed results. For example, Song and Caruso’s (1996) investigation of the differences in scoring of ESL students’ essays by NES and NNES instructors indicated that NNES teachers were stricter with holistic scores of writing than their NES peers, but there was no statistically significant difference in analytical scoring between the two groups of teachers. However, some research has reported different findings. Connor-Linton (1995) recruited 26 NES and 29 NNES teachers to compare their grading of Japanese EFL learners’ writing. No significant difference between the two groups of teachers’ holistic scores was reported. Aside from the quantitative scoring, they also examined NES and NNES teachers’ qualitative justifications for their scores and found that NES teachers paid more attention to the quality of high-order dimensions of writing, whereas their NNES colleagues focused more on linguistic accuracy. Following Connor-Linton’s study, Shi (2001) invited 23 NES and 23 NNES teachers to rate 10 Chinese EFL learners’ writing. The study also reported that NES and NNES teachers did not differ significantly in holistic scoring. However, the analysis of teachers’ reasons for their scoring showed that NES teachers put emphasis on students’ linguistic quality, while NNES teachers were more concerned with content and general organization.

In addition, researchers have investigated NES and NNES teachers’ assessment of errors in L2 writing (e.g., Porte, 1999; Hyland and Anan, 2006; Rao and Li, 2017). For example, Porte (1999) undertook a study to address the differences between the two groups of teachers in terms of evaluating errors. In his study, 14 NES and 16 NNES teachers were recruited to respond to a series of sentences in L2 learners’ writing, each of which contained an error. This study reported a gap in error identification between the two groups of teachers. NES teachers demonstrated a more lenient attitude toward errors than NNES teachers. To discover whether there were differences in teachers’ perceptions of error gravity in a different L2 context, Hyland and Anan (2006) included three groups: NNES teachers, NES teachers, and native non-teachers in their study. The participants were required to pick out errors in a piece of English writing drafted by a Japanese EFL learner. They also found that NNES teachers were more severe than their NES peers in identifying errors in L2 writing. More recently, Rao and Li’s (2017) study, in the Chinese EFL context, examined NES and NNES teachers’ assessment of errors in writing samples by a cohort of Chinese EFL learners. Consistent with previous studies, they found that compared with NES teachers, NNES teachers showed less tolerance of students’ linguistic errors in writing. Furthermore, they attributed the differences between NES and NNES teachers’ identification of errors to four factors: Cultural beliefs, educational background, teaching style, and English proficiency. Based on these studies, researchers, to date, seem to reach a consensus that NES teachers tend to be more lenient of language errors than their NNES peers.

In summary, although the above studies regarding L2 teachers’ written feedback practices as well as NES and NNES teachers’ assessment in L2 writing are inspirational and thought-provoking, there are still two research gaps. Firstly, the majority of studies on teachers’ practices about written feedback are conducted in L2 contexts outside of mainland China, so little is known about how L2 teachers implement written feedback in this context (Mao and Crosthwaite, 2019; Yu et al., 2020). The findings of studies in other L2 contexts may be not generalized, all the time, to the mainland Chinese EFL context. More importantly, to our knowledge, no study until now has systematically investigated and compared written feedback practices by NES and NNES teachers, who are born, bred, and educated in very different sociocultural contexts. As mentioned previously, written feedback is not a decontextualized activity, but a social practice in which teachers’ cultural and language backgrounds play an important role (Lee, 2014; Bitchener and Storch, 2016; Hyland and Hyland, 2019). Therefore, such a comparative study is needed. Their different practices shown by our study possibly enable the two groups of teachers to cooperate with and learn from each other in terms of providing feedback, which may improve their expertise of feedback provision, enhance the effectiveness of their written feedback, and contribute to their students’ EFL writing learning. To close the gaps, this study intended to address the following research question: *How do NES and NNES teachers implement written feedback on their Chinese EFL learners’ writing?*

## The Study

### Participants and Research Context

This study was conducted in a central city in mainland China. The choice of this city was practically motivated. Specifically, the first author was born and grew up in this city, so it was more likely for him to gain access to universities in his hometown than other cities in China. After the ethics application was approved at The University of Auckland, New Zealand, the first author of this study drew upon his personal network to contact the potential universities, and finally had access to three universities with consent.

Our study employed a purposive sampling strategy to recruit the participants, as it is a widely used approach for the selection of participants “who can provide rich and varied insights into the phenomenon under investigation so as to maximize what we can learn” (Dörnyei, 2007, p. 126). Thus, the following criteria were taken into account (see Table 1).

**TABLE 1:** Criteria for participant selection.

Criteria	Descriptions
(1) First language	Chinese L1 and English L1 speakers
(2) Experience in teaching EFL writing	At least 2-year teaching experience in EFL writing
(3) Academic qualifications	Bachelor’s degree or above
(4) Majors	English-related majors such as literature, linguistics/applied linguistics, translation, and TESOL
(5) Others	(1) Teaching EFL writing during data collection; (2) Being available and willing to participate in this study

To approach the teacher participants, Deans of the relevant Faculties in the three universities were contacted and the aims of this study were explained in detail. With their permissions, the selection criteria, participant information sheets, and consent forms were emailed to all the EFL teachers in the faculty through the Dean’s secretary in each university. 11 EFL writing teachers responded (five NES and six NNES teachers). These teachers were met individually and briefed on the research purposes, procedures, and their role in this study, and they were assured that their identities would be protected and kept confidential. Eventually, eight teachers (four NES and four NNES teachers) agreed to participate and signed consent forms. Their demographic information is presented in Table 2.

**TABLE 2:** Profile of teacher participants.

Names (pseudonym)	NES vs. NNES	Countries of origin	Countries where they obtained higher degree	English proficiency	Experience in teaching EFL writing	Academic qualifications	Majors
Jason	NES	US	US	—	4 years	Bachelor	Linguistics
George	NES	US	US	—	3 years	Master	TESOL
Bruce	NES	US	US	—	6 years	Master	Literature
Christine	NES	US	US	—	4 years	Bachelor	Literature
Yan	NNES	China	China	IELTS 7.0	8 years	Master	TESOL
Juan	NNES	China	China	IELTS 7.5	5 years	Master	Literature
Han	NNES	China	China	IELTS 7.5	3 years	Master	Applied linguistics
Qin	NNES	China	China	IELTS 7.5	4 years	Master	Linguistics and applied linguistics

Our participants were EFL writing teachers of English major sophomores. During the data collection, all the English major sophomores in these teachers’ universities were enrolled in *English Writing Course*, which aimed to improve students’ basic knowledge in English writing, enhance their writing proficiency in different genres, and help them pass the TEM-4^1^. The course was delivered weekly with two, 45-min class periods in a 16-week semester, and the class size was around 30 students. In such a writing course, students were required to complete four or five pieces of writing in or after class, which were used to assess their course performance. Commonly, the topics for the writing tasks were selected from the past TEM-4 test battery, and students were asked to compose their writing with approximately 200 words within 40 min by themselves. After students had finished their first drafts of writing, the teachers scored them based on the writing rubrics of TEM-4 and provided students with written feedback. There were no strict university guidelines on teachers’ written feedback provision. After that, students revised their writing according to their teachers’ feedback within a week.

Apart from the eight teacher participants, 80 second-year students (11 males and 69 females) from the eight teachers’ classes participated in this study voluntarily. They were students in English major and the collection of data from such English-major students was due to the fact that only English-major students are offered English writing course separately in many Chinese universities (Zhang, 2016; Li et al., 2020). All these students were Mandarin Chinese speakers and their ages ranged from 19 to 21 with, on average, 11 years of English learning experience. They shared similar educational experience. That is, they received their primary and secondary education in mainland China without overseas learning experience. Based on their English test scores at the end of last semester and discussions with the eight teachers, such students were considered as pre-intermediate/intermediate EFL learners.

### Data Collection

In our study, we gathered pertinent information on how NES and NNES teachers provided written feedback mainly from their Chinese EFL students’ writing samples that showed teacher written feedback. In addition, some complementary data were collected from documents such as teachers’ teaching plans, writing syllabus, and TEM-4 writing rubrics, which could provide contextual information about the present study.

Unlike many previous studies, which used the texts with a same topic by a particular group of students to compare NES and NNES teachers’ writing assessment practices, in our study we gathered writing samples, which were written assignments from each teacher’s writing course. Such an approach to collecting data was more authentic and uncontrolled, which contributes to a better understanding of teachers’ practices regarding written feedback in a naturally occurring situation. A total of 80 writing samples were collected for data analysis (10 per teacher). These writing samples were drafted by English major sophomores within 40 min in class, the length of which ranged from 150 to 200 words. During the writing process, students were forbidden to use any external resources to assist their writing.

Given that these 80 writing texts were collected from different teachers’ writing class and we did not interfere their assignment of tasks, it was difficult to collect writing samples with a same topic from them. In order to ensure the task difficulty, two measures were adopted. We, firstly, collected writing texts with a same genre, that is, argumentative writing, which is a commonly used assessment for L2 learners’ writing proficiency (Li et al., 2020). In the Chinese tertiary EFL context, students tend to be asked to write argumentative essays in various large-scale standardized English proficiency tests such as IELTS, TOEFL, College English Test Band 4 and 6 (CET-4 and 6), and Test for English Majors Band 4 and 8 (TEM-4 and 8) (Huang and Zhang, 2020). More importantly, the topics for these collected texts came from past TEM-4 papers. As TEM-4 is a large-scale and well-established test, its writing topics are of high reliability and validity. Specifically, the topics tend to be designed as general and drawn upon students’ daily life, so they are considered familiar and fair to every student, and their task difficulty is guaranteed to be consistent (Zheng and Cheng, 2008; Teng and Zhang, 2020).

### Data Analysis

To understand NES and NNES teachers’ normal practices regarding written feedback on their Chinese EFL learners’ writing, we scrutinized the feedback instances in different locations of students’ writing, such as between lines, in the margins, and at the end of writing. Feedback points, in our study, were defined as the written interventions by teachers (Hyland, 2003) and the analysis was based on meaningful units, which might be in the form of a symbol, word, phrase, or sentence (Yu and Lee, 2014). In the practical coding, teachers’ written feedback was analyzed in terms of feedback focus, feedback strategy, and feedback scope.

Firstly, all the identified feedback points were categorized according to what they targeted in focus, that is, whether feedback focused on global or local issues (global feedback vs. local feedback). We adapted the scheme originally proposed by Storch and Tapper (2000) and Alkhatib (2015) to code feedback focus (see Table 3).

**TABLE 3:** Coding scheme for feedback focus.

Focus	Subcategory	Example
Language	Grammar and vocabulary	• ‘Instead of bring harm…’ → *‘bringing’* • ‘Cell phone plays…’ → *‘cell phones’* • ‘Putting a pollution tax’ → *‘imposing’*
Content	Clarity, adequacy and relevance	• *What do you mean by this sentence?* • *Give another reason to support your idea* • *The supporting details in paragraph two are irrelevant to the topic sentence*
Organization	The overall structure, cohesion, coherence and paragraphing	• *There is no topic sentence in this paragraph* • *Add “firstly” here* • *One paragraph should develop one idea*

**Examples are retrieved from the feedback provided by participating teachers and are put in italics.**

Next, the feedback points were further analyzed with regard to feedback strategies. According to the realization ways of direct and indirect feedback strategies introduced in the literature review, we coded the global and local written feedback into direct and indirect feedback. Table 4 presents the different formulations of direct and indirect written feedback.

**TABLE 4:** Realizations of direct and indirect written feedback.

Strategy	Realization	Example
*Local feedback*		
Direct feedback	(1) Presenting the correct answers directly (2) Crossing out the redundant or erroneous items (3) Adding the omitted items	• *Using cell phone is very popular among young people… (phones);* • *How to establish and maintain friendship online?* • *An increasing number of people use phones*,^∧^ *they have a profound influence on our society.* (^∧^*and)*
Indirect feedback	(1) Indicating errors without meta-linguistic clues (2) Indicating errors with meta-linguistic clues	• *Writing letters bring some benefits to us…* • *It is a good way to study some practical knowledge… (word choice)*
*Global feedback*		
Direct feedback	(1) Making overt corrections (2) Offering feasible suggestions	• ^∧^*Imposing a pollution tax on private car drivers benefits environmental protection (adding a topic sentence).* • *Another reason for not making friends online is needed. For example, it poses threats to people’s safety.*
Indirect feedback	(1) Indicating problems only	• *A lack of concluding paragraph These details are irrelevant to the topic sentence of this paragraph.*

**Examples are retrieved from the feedback provided by participating teachers and are put in italics.**

Finally, after coding feedback focus and strategies, we calculated the total amount of written feedback provided by each teacher as well as that of local/global feedback, and direct/indirect feedback. In this way, we could determine whether each teacher provided written feedback in a comprehensive or selective way.

Overall, the process of analyzing teacher written feedback was recursive and iterative rather than linear in order to gain a comprehensive understanding of the similarities as well as differences between NES and NNES teachers’ feedback practices. We analyzed teachers’ feedback points quantitatively and qualitatively. From the quantitative analysis, the descriptive data such as frequencies and percentages were obtained. Chi-square (χ^2^) analyses were conducted to detect the significant difference of the distribution of feedback points. Furthermore, independent samples *t*-tests were administered to examine the group differences. Before conducting such tests, we checked whether the data were normally distributed and found that they met the requirement of normal distribution that the standardized skewness values are between 0 and ±3.0 and standardized values of kurtosis values do not exceed ±8.0 (Field, 2009). Aside from the quantitative data, the feedback instances were processed qualitatively to depict a picture about what errors/problems teachers actually focused on and how they delivered their feedback to their students in practice. The qualitative analysis could triangulate with the quantitative data, and make detailed explanations as well as add more information to the quantitative data.

To maintain the trustworthiness of the findings, an EFL writing teacher who did not participate in our study and had obtained her master’s degree in applied linguistics was invited to be a co-coder. At first, 20% of the collected writing samples (16/80 samples) was selected randomly and coded by her and the first author of this study independently. The intercoder reliability for coding each category was: Feedback focus (*r* = 0.94), feedback scope (*r* = 0.96), and feedback strategy (*r* = 0.91). To further improve the coding reliability, the disagreements in coding were discussed until they were resolved and then the first author coded the rest of data independently.

## Findings

### NES and NNES Teachers’ Written Feedback Focus

As our analysis showed, the four NES teachers provided feedback on both local and global issues in the collected 40 texts (see Table 5), but there was a significant difference in the distribution of written feedback across the two categories (χ^2^ = 111.57, *df* = 1, *p* = 0.000). In total, the participants offered 495 feedback points in their students’ writing, among which 113 (22.83%) on grammar, 17 (3.43%) on vocabulary, 173 (34.95%) on content, and 192 (38.79%) on organization. Notably, over 70% of NES teachers’ written feedback was delivered to global issues. This indicates that they had a stronger focus on global issues when responding to students’ writing.

**TABLE 5:** NES and NNES teachers’ written feedback on local and global issues.

Teacher	Local issues		Global issues		Total
	Grammar	Vocabulary	Content	Organization	
**NES teachers**					
George	23 (18.55%)	5 (4.03%)	46 (37.10%)	50 (40.32%)	124
Jason	36 (28.13%)	5 (3.91%)	44 (34.38%)	43 (33.59%)	128
Bruce	29 (22.48%)	4 (3.10%)	44 (34.11%)	52 (40.31%)	129
Christine	25 (21.93%)	3 (2.63%)	39 (34.21%)	47 (41.23%)	114
Total	113 (22.83%)	17 (3.43%)	173 (34.95%)	192 (38.79%)	495
**NNES teachers**					
Juan	158 (68.10%)	35 (15.28%)	26 (11.35%)	10 (4.37%)	229
Yan	130 (67.36%)	44 (22.80%)	14 (7.25%)	5 (2.59%)	193
Qin	131 (66.84%)	39 (19.90%)	10 (5.10%)	16 (8.16%)	196
Han	118 (67.82%)	41 (23.56%)	8 (4.60%)	7 (4.02%)	174
Total	537 (67.80%)	159 (20.08%)	58 (7.32%)	38 (4.80%)	792

Furthermore, their feedback on content could be broken into three subcategories: Clarity (72/173, 41.62%), relevance (28/173, 16.18%), and adequacy (73/173, 42.20%) with a significant difference in the number of written feedback points across the three subcategories (χ^2^ = 22.90, *df* = 2, *p* = 0.000). This suggests that more comments were given to clarity and adequacy than relevance. The following examples illustrate NES teachers’ written feedback on clarity and adequacy.

Example 1

**Table:** 

**Student text**	*And there also be some measures to try to make education more academically rigorous and to tackle a culture in the education establishment…*
**Christine’s feedback**	What’s the meaning of this sentence?

Example 2

**Table:** 

**Student text**	*The reason why they should not taxed is quite simple. The majority of private car owners are ordinary people who go to work by car for their most convenience…*
**George’s feedback**	Only one reason is provided. Please add another different reason to support your claim.

As Example 1 shows, Christine used a feedback comment to highlight a problem related to clarity in a student’s writing. Such a practice could probably cultivate the student writer’s awareness of the need to convey meaning clearly and then might help him/her make progress in this aspect. Example 2 is feedback on adequacy. According to this example, George commented on the reasons provided in writing. He emphasized that students should formulate at least two different reasons to develop their ideas. Thus, he offered the feedback, asking the student writer to add another one reason. Apart from the number of reasons to develop ideas, NES teachers also stressed that topic sentences should be developed adequately with examples or reasoning. That is to say, students should elaborate on topic sentences sufficiently with details rather than simply formulate topic sentences at the beginning of paragraphs. The following example is a comment by Bruce to point out the limited development of a topic sentence. In Example 3, he first identified the problem and then suggested the student how to resolve it.

Example 3

**Table:** 

**Student text**	*My arguments for this point are listed as follows. The main reason for my view is that many tourists have high education and environmental awareness. They have a good sense of environmental protection.*

**Table:** 

**Bruce’s feedback**	Lack of details to support the topic sentence. You can add an example “they do not throw rubbish when visiting some places of interest” to illustrate your idea.

Similarly, the NES teachers’ 192 feedback points on organization were further analyzed and subdivided into four categories: Overall structure, cohesion, coherence, and paragraphing. Based on a chi-square test, the distribution of the four subcategories reached statistical significance (χ^2^ = 48.38, *df* = 3, *p* = 0.000). Specifically, they gave the most feedback on cohesion when assessing students’ organization of writing (79/192, 41.15%). The remaining three subcategories were ranked as follows: Overall structure (64/192, 33.33%), coherence (25/192, 13.02%), and paragraphing (24/192, 12.50%). According to the qualitative analysis, NES teachers’ feedback on cohesion mainly focused on two aspects: conjunctions and references. Example 4 shows Christine’s attention to cohesion realized by the use of conjunctions. She added a conjunction “firstly” to start the paragraph in order to signify that this was the first reason, which made the two different reasons tied clearly and smoothly. Another instance concerns students’ use of references, which helped them establish the anaphoric relationship in writing. In such an example, George underlined the pronoun “it” and asked the student writer to clarify what “it” here referred to.

Example 4

**Table:** 

**Student text**	^∧^*As an old saying goes: all work and no play makes Jack a dull boy. Obviously, parents take children out of school for holiday not only broadens their horizons but also help them relax both mentally and physically…*
**Christine’s feedback**	(At the beginning of the paragraph) ^∧^Firstly

Example 5

**Table:** 

**Student text**	*It is obvious that it can reduce pollution and is good for our environmental protection…*
**George’s feedback**	What does it here refer to? Specify.

Regarding the NNES teachers’ written feedback focus in practice, 792 written feedback points were generated by them, and these feedback points addressed both local and global issues as well. Specifically, of the 792 written feedback points, 696 feedback points (87.88%) were given to errors in the local dimensions, while 96 (12.12%) to the global areas (see Table 5) and the number differed significantly (χ^2^ = 454.55, *df* = 1, *p* = 0.000). This suggests that NNES teachers showed much more concern with local issues when giving written feedback. Such a finding does not align with their NES peers’ practices, in which they marginalized local issues and were more sensitive to global issues.

When it comes to the nature of global issues, content (58/96, 60.42%) received more attention than organization (38/96, 39.58%). In line with NES teachers, their NNES peers’ feedback on content was subdivided into clarity (48/58, 82.76%), adequacy (6/58, 10.34%), and relevance (4/58, 6.90%), indicating that NNES teachers’ feedback on content primarily targeted issues related to clarity. As for their feedback on organization, it comprised four subcategories: Cohesion (26/38, 68.42%), overall structure (9/38, 23.68%), coherence (1/38, 2.63%), and paragraphing (2/38, 5.26%), which suggests that NNES teachers paid more attention to cohesion than other dimensions. Given the amount of feedback on clarity and cohesion provided by NES and NNES teachers, it appeared that they shared the similar foci when delivering feedback to their Chinese EFL learners’ content and organization in writing.

In terms of the nature of local issues where written feedback was provided on writing samples, grammatical errors (537/696, 77.16%) took precedence over lexical errors (159/696, 22.84%). Moreover, the observation of their written feedback revealed that NNES teachers corrected different types of language errors. As evidenced by Example 6, Juan provided feedback on different grammatical errors (e.g., plural forms, parts of speech, and articles) and word choice.

Example 6

**Table:** 

**Student text**	*First, using phone to send information is more convenience. Many people complain that writing the letter wastes time. Young people just type on phones and send the message immediately, which saves much time and energy.* *Second, using phoneis fitted into young people. Today, young people have a large need to communicate with each other. Letter writing have trouble in sending too many information.*
**Juan’s feedback**	Phone → phones; convenience → convenient; the → a; the message → messages; is fitted into → is suitable for; large: wrong word; have → has; many → much

To sum up, this section discussed the details of the two groups of teachers’ feedback practices regarding focus. According to independent samples *t-*tests, significant differences existed between NES and NNES teachers’ written feedback in terms of the number of feedback points on language (*t* = −20.59, *p* = 0.000, *d* = −4.60), content (*t* = 11.10, *p* = 0.000, *d* = 2.48), and organization (*t* = 17.19, *p* = 0.000, *d* = 3.84). Such results suggest that NES and NNES teachers’ written feedback focus was significantly different. Specifically, when giving feedback in practice, NES teachers had a stronger focus on content and organization (global issues), whereas their NNES peers gave much more importance to language (local issues).

### NES and NNES Teachers’ Written Feedback Scope

With regard to the two groups of teachers’ written feedback scope in practice, as shown by Table 5, both provided written feedback targeting a range of global and local issues. As a result, they did not focus on only a limited number of errors in responding to their students’ writing. Furthermore, according to Table 6, the four NES teachers offered a great deal of feedback. Averagely, each student’s writing sample received 12.4 feedback points. Given the total number of words per written text (150–200), such an average number was large. This demonstrates that NES teachers corrected Chinese EFL learners’ writing comprehensively actually. As for their NNES colleagues, Table 6 shows that they also provided a large amount of written feedback. The average number of feedback points that the NNES teachers provided to each writing sample was high relative to the length of the writing (150–200 words), which suggests that NNES teachers offered feedback in a comprehensive way as well when marking their students’ writing.

**TABLE 6:** The amount of written feedback provided by NES and NNES teachers.

Participant	Amount		
	Total	Average	Range
**NES teachers**			
George	124	12.4	9–15
Jason	128	12.8	11–15
Bruce	129	12.9	7–16
Christine	114	11.4	8–16
Total	495	12.4	7–16
**NNES teachers**			
Juan	229	22.9	18–29
Yan	193	19.3	9–27
Qin	196	19.6	15–25
Han	174	17.4	13–25
Total	792	19.8	9–29

Although NES and NNES teachers provided written feedback in a detailed and extensive way, they did not necessarily correct each error in their students’ writing. They left aside some errors intentionally or inadvertently. As Example 7 demonstrates, Bruce did not cross out the preposition “to,” which was redundant. Similarly, in Example 8, the student writer wrote a run-on sentence, but Juan did not give any feedback to it.

Example 7

**Table:** 

**Student text**	*Consequently, I totally disagree that tourism will harm to our nature environment…*
**Bruce’s feedback**	No feedback

Example 8

**Table:** 

**Student text**	*In ancient times, technology was underdeveloped, handwriting is a way of communicating with people of culture…*
**Juan’s feedback**	No feedback

In conclusion, both teachers shared the similar actual practices in this theme. That is, they offered comprehensive written feedback. In spite of this, the independent samples *t*-test showed that the number of feedback points produced by these two groups of teachers was significantly different with a large effect size (*t* = −9.48, *p* = 0.000, *d* = −2.12). This indicates that NNES teachers offered significantly more feedback points than their NES counterparts, even though both provided written feedback comprehensively.

### NES and NNES Teachers’ Written Feedback Strategies

Table 7 reveals that NES and NNES teachers concurrently adopted direct and indirect feedback strategies to deliver written feedback to their students. However, there is a discrepancy in regard to the distribution of direct and indirect feedback points provided by the four NES and NNES participating teachers. According to Table 7, the NES teachers offered 231 direct and 254 indirect feedback points^2^. On average, they provided 5.8 direct and 6.4 indirect feedback points on each student’s writing, respectively. Moreover, no significant difference was found in the distribution of direct and indirect written feedback points (χ^2^ = 1.09, *df* = 1, *p* = 0.296). Such a result shows that the NES teachers provided similar amount of direct and indirect feedback in response to their students’ written assignments. As for their NNES peers’ actual practices regarding feedback strategies, 450 direct and 342 indirect feedback points were produced by the four NNES teachers, with the mean average number of direct and indirect feedback points totaling 11.3 and 8.6 in each collected written text. According to a chi-square test, there was a significant difference in the number of direct and indirect feedback points (χ^2^ = 14.73, *df* = 1, *p* = 0.000), indicating that NNES teachers utilized direct feedback more frequently than indirect feedback to mark Chinese EFL learners’ writing.

**TABLE 7:** NES and NNES teachers’ written feedback strategies.

Participant	Strategies		
	Direct feedback	Indirect feedback	Total
**NES teachers**			
George	55 (45.83%)	65 (54.17%)	120
Jason	60 (47.62%)	66 (52.38%)	126
Bruce	62 (48.82%)	65 (51.18%)	127
Christine	54 (48.21%)	58 (51.79%)	112
Total	231 (47.63%)	254 (52.37%)	485
**NNES teachers**			
Juan	120 (52.40%)	109 (47.60%)	229
Yan	99 (51.30%)	94 (48.70%)	193
Qin	135 (68.88%)	61 (31.12%)	196
Han	96 (55.17%)	78 (44.83%)	174
Total	450 (60.61%)	342 (43.18%)	792

Furthermore, the analysis of teachers’ actual practices on their students’ writing drew a complex and nuanced picture regarding their employment of feedback strategies. Despite NES and NNES teachers combining both direct and indirect feedback in practice, their choice of direct and indirect feedback strategies was mediated by the types of errors/problems. As Table 8 shows, NES and NNES teachers were used to employing direct feedback to correct local issues (NES: 87/130, 66.92%; NNES: 427/696, 61.35%), while they provided significantly more indirect feedback to address global issues (NES: 211/355, 59.44%; NNES: 73/96, 76.04%). The following examples demonstrate how teachers provided direct and indirect feedback to address their students’ local and global issues, respectively.

**TABLE 8:** NES and NNES teachers’ indirect and direct feedback across error types.

Participant	Local issues		Global issues		Total
	Direct	Indirect	Direct	Indirect	
**NES teachers**					
George	19 (15.83%)	9 (7.50%)	36 (30.00%)	56 (46.67%)	120
Jason	27 (21.43%)	14 (11.11%)	33 (26.19%)	52 (41.27%)	126
Bruce	25 (19.69%)	8 (6.30%)	37 (29.13%)	57 (44.88%)	127
Christine	16 (14.29%)	12 (10.71%)	38 (33.93%)	46 (41.07%)	112
Total	87 (17.94%)	43 (8.87%)	144 (29.69%)	211 (43.51%)	485
**NNES teachers**					
Juan	112 (48.91%)	81 (35.37%)	8 (3.49%)	28 (12.23%)	229
Yan	93 (48.19%)	81 (41.97%)	6 (3.11%)	13 (6.74%)	193
Qin	129 (65.82%)	41 (20.92%)	6 (3.06%)	20 (10.20%)	196
Han	93 (53.45%)	66 (37.93%)	3 (1.72%)	12 (6.90%)	174
Total	427 (53.91%)	269 (33.96%)	23 (2.90%)	73 (9.22%)	792

As revealed by Examples 9 and 10, teachers presented direct feedback to linguistic errors for students. In the first example, Jason wrote the correct form and deleted the redundant item. In the latter example, Yan provided the correct answer and added the omitted item. It seemed that NES and NNES teachers employed at least two techniques presented in Table 4 to formulate direct feedback on linguistic errors.

Example 9

**Table:** 

**Student text**	*I telled my sufferings to her and I need not to worry about being laughed at…*
**Jason’s feedback**	Telled → told; to → to

Example 10

**Table:** 

**Student text**	*Volunteer work become more and more popular around the world. There are over tens of millions of people have volunteered to help those in need…*
**Yan’s feedback**	Become → becomes; tens of millions of people have*…* → tens of millions of people ∧ who have*…*

Examples 11 and 12 show how teachers used indirect feedback to address students’ problems in global areas. According to the two examples, NES and NNES teachers just highlighted and identified the problems for their students without any corrections or suggestions. Such a practice could make students aware of their problems, but they needed to engage themselves in resolving them. In Example 11, Bruce found that the student writer failed to provide enough details to support the topic sentence, so he indicated the problem by making a comment “no details.” By doing so, the student’s attention could be directed to such a problem. As such, the student probably realized his/her problem in this regard. Likewise, in Example 12, Juan highlighted the problem with a metalinguistic description “a lack of topic sentence,” which might enable the student writer to understand this problem in his/her writing.

Example 11

**Table:** 

**Student text**	*Compared with other industries, tourism is more promising on the account of that it never uses that kind of resources like fossil oil and raw materials.*
**Bruce’s feedback**	No details.

Example 12

**Table:** 

**Student text**	*Secondly, when we spend more time on phones, we will know less about how to write a letter…*
**Juan’s feedback**	Where’s the topic sentence?

## Discussion

### NES and NNES Teachers’ Practices Regarding Feedback Focus

In our study, NES and NNES teachers showed contrasting feedback focus in their actual practices. Specifically, NES teachers put more emphasis on global issues, whereas their NNES peers were more aware of local issues while providing Chinese EFL learners with written feedback. The finding that NNES EFL teachers paid much more attention to linguistic errors can be observed in previous studies (e.g., Lee, 2008, 2009, 2011; Alkhatib, 2015; Mao and Crosthwaite, 2019), in which such teachers provided written feedback on language predominantly.

Our finding that NES and NNES teachers presented different orientations in focus when providing written feedback (global issues vs. local issues) aligns with that of the existing studies, which have compared NES and NNES teachers’ teaching practices in other fields including general English teaching (meaning vs. accuracy) (Arva and Medgyes, 2000), grading EFL writing (high-order dimensions of writing vs. language) (Connor-Linton, 1995), and assessing L2 learners’ oral English proficiency (content vs. grammar) (Zhang and Elder, 2011). In a sense, our study extends the current investigations concerning NES/NNES teachers’ pedagogical practices to the sphere of teacher written feedback in L2 writing.

Native English-speaking and NNES teachers’ gap in feedback focus is hardly surprising, which may be ascribed to several plausible factors. One is probably associated with NES and NNES teachers’ teaching styles. As for NES teachers, their teaching style is reported to be characterized by being global, open, and communication-oriented (Rao, 2002, 2010). With such a teaching style, they probably regard students’ writing as a whole, evaluate it from a global perspective, and emphasize its communicative purpose, instead of mainly focusing on linguistic issues in writing. In comparison, NNES teachers’ teaching style is concrete-sequential, closure-oriented, and rule-based (Melton, 1990; Rao and Li, 2017; Su, 2019). This type of teaching style values accuracy and linguistic details, leading to NNES teachers’ emphasis on students’ language use (Rao, 2002), which may account for their meticulous correction of students’ linguistic errors in feedback provision.

Another factor may be related to NES and NNES teachers’ English proficiency. Although the NNES teachers in our study had high level of English proficiency, they are naturally inferior to their NES peers with regard to the mastery of English and the proficient use of English (Rao and Yuan, 2016). As an important variable, English proficiency influences teachers’ tolerance of English language errors (Hughes and Lascaratou, 1982; Rao and Li, 2017). Thus, NES teachers are of higher tolerance of language errors, which may enable them to shift their attention from linguistic level to high-order dimensions of writing when providing their Chinese EFL students with written feedback on writing. In comparison, NNES teachers are not able to use English language as flexibly and proficiently as their NES peers. Thus, they tend to be stricter with language errors (Hyland and Anan, 2006; Rao and Li, 2017), which probably makes NNES teachers pay much more attention to linguistic errors in feedback provision.

Finally, such a difference may be associated with the two groups of teachers’ prior experience in language learning. With the popularity of the communicative teaching approach in western countries, NES teachers are probably educated to concentrate their attention on meaning and comprehension, with less importance attached to language in language learning (Rao and Li, 2017). In contrast, NNES teachers have had different language learning experience in L2 classrooms, where “language is more likely to be an object in its own right with a concomitant focus on form” (Zhang and Elder, 2011, p. 43). In such classrooms, NNES teachers are instructed by the grammar-translation approach in English learning, which prioritizes discrete grammatical points and stresses linguistic accuracy (Rao, 2002; Jin and Cortazzi, 2006). They are used to focusing on grammatical errors in each sentence of writing, which may explain their little attention being given to global issues when providing feedback.

In spite of the significant difference in the focus of their feedback, NES and NNES teachers’ written feedback practices shared the commonality in the nature of local feedback. To be specific, when dealing with local issues, they paid more attention to grammatical errors than lexical errors. This finding is in line with that of prior studies (e.g., Montgomery and Baker, 2007; Junqueira and Payant, 2015; Mao and Crosthwaite, 2019). Such a finding is probably due to much more grammatical errors actually occurring in students’ writing. In addition, it may also be attributed to the properties of grammatical and lexical errors. Many grammatical errors (e.g., articles, agreement, voice, plurality/singularity, tense…) tend to be grouped into treatable errors, which are rule-governed, while lexical errors are commonly regarded as untreatable errors, which are relatively difficult to explain by rules (Ferris, 2002). Thus, it is comparatively difficult for teachers to correct lexical errors, which may attract less feedback targeting them.

### NES and NNES Teachers’ Practices Regarding Feedback Scope

Our analysis of feedback showed that both the two groups of teachers adopted a comprehensive approach to feedback provision, focusing on an array of errors in Chinese tertiary EFL writing classrooms. This finding is not surprising and may be because of Chinese traditional culture. In such a culture, teachers are defined as “persons who propagate doctrines, impart professional knowledge, and resolve doubts” (Li, 2014, p. 4), so they shoulder a highly responsibility for students’ learning development. To fulfill their responsibility, they need to provide feedback comprehensively. Otherwise, they would be considered irresponsible if they provided focused feedback, which targets a limited number of errors and does not attend to others.

Our finding that Chinese EFL teachers provided written feedback comprehensively is consistent with prior literature on L2 teachers’ feedback practices in different contexts such as Saudi (Alkhatib, 2015) and Hong Kong (Lee, 2008, 2011). In this sense, it appears that comprehensive feedback has become a ubiquitous instructional practice in L2 classrooms across different EFL contexts and countries. Furthermore, our finding advances the current studies in this line. Specifically, such studies mainly focus on NNES EFL teachers’ practices regarding feedback scope. However, our study also paid attention to their NES counterparts, finding that they also provided written feedback in a comprehensive way.

Interestingly, although both NES and NNES teachers marked their students’ writing in an extensive way, NNES teachers gave significantly more feedback points than NES teachers. There are two potential explanations for this finding. One is that NES teachers may be more lenient about errors than NNES teachers, as observed in some prior studies (Porte, 1999; Hyland and Anan, 2006; Rao and Li, 2017). In these studies, NES teachers tended to hold a tolerant attitude toward errors, which may allow them to ignore some errors. The other possible explanation is the different focus of NES and NNES teachers in feedback provision. The analysis of feedback suggested that NES teachers placed more emphasis on problems related to content and organization, whereas NNES teachers, when providing written feedback, paid more attention to local issues. Therefore, it is unsurprising that the number of linguistic errors is larger than that of global issues, since errors in relation to language can appear in each sentence.

### NES and NNES Teachers’ Practices Regarding Feedback Strategies

In our study, Chinese university EFL writing teachers combined both direct and indirect feedback strategies when providing feedback, irrespective of their L1. However, NES teachers offered the similar number of direct and indirect feedback points, while their NNES peers gave much more direct feedback than indirect feedback. The finding that EFL teachers delivered their written feedback both directly and indirectly is commonly reported in previous studies (e.g., Lee, 2011; Alkhatib, 2015; Zheng and Yu, 2018; Mao and Crosthwaite, 2019). In such studies, L2 writing teachers did not use direct or indirect feedback alone. Such a practice also corresponds to the principle posited by Bitchener and Storch (2016) that direct and indirect feedback should be combined in use because neither of them is the best for learning.

More importantly, the two groups of teachers’ use of feedback strategies was strongly mediated by the types of errors/problems. Specifically, when responding to local issues (i.e., linguistic errors), they used direct feedback more frequently. This finding parallels that of previous studies (e.g., Lee, 2008, 2011; Zheng and Yu, 2018). However, such a finding does not concur with what Alshahrani and Storch (2014) have found. In their study, EFL writing teachers used more indirect feedback to treat local issues. The inconsistent result revealed by their study is probably related to the school policy, which compelled their participants to provide written feedback indirectly.

When it comes to problems in content and organization, both NES and NNES teachers used more indirect feedback to address them. Specifically, they tended to highlight problems for their students without making any corrections or suggestions for improvement. Such a tendency is also observed in Junqueira and Payant’s (2015) study and may be explained by the following possible reasons. Firstly, in comparison with linguistic errors, it is more difficult and challenging for teachers to address issues related to content and organization directly because it requires more extensive information. In this situation, indirect feedback may be a more suitable strategy to solve such problems.

Furthermore, different from linguistic errors, global issues are, in nature, neither clearly right nor wrong. Thus, it is very likely that there are several solutions to a particular global problem. It is probably unsuitable for teachers to employ direct feedback to address it. If they do so, it appears that they appropriate their students’ own texts (Hyland and Hyland, 2001).

Overall, NES and NNES tertiary writing teachers’ written feedback practices in the Chinese EFL context created an impression that the practices were teacher-centered and teacher-directed, while their students fail to assume full responsibility to identify and correct their own errors/problems in writing. This could be evidenced by teachers’ comprehensive approach to feedback provision and provision of a great many direct feedback points. Teacher-dominated pedagogical practices in the Chinese context are not unexpected and may be associated with Chinese traditional culture, in which teachers have an absolute authority and can decide what and how to teach, while their students are merely passive knowledge recipients (Bao, 2019; Sheng, 2019). Interestingly, brought up and instructed in a very different educational culture, which emphasizes students’ role and agency in learning, NES teachers in our study showed similar teaching practices as their NNES peers. This is quite understandable. As the outsiders of Chinese culture, they need to factor it into their pedagogical decision-making and adopt culturally appropriate teaching practices in order to avoid cultural conflicts (Ma, 2012; Rao and Yuan, 2016). This suggests that teacher written feedback is a teaching behavior influenced by cultural norms and expectations within which teachers operate, rather than a practice in a vacuum (Lee, 2014; Bitchener and Storch, 2016; Storch, 2018).

## Conclusion

As a crucial pedagogical affordance in L2 writing classrooms, teacher written feedback benefits L2 learners’ writing development. Embedded in the mainland Chinese EFL context, our study explored NES and NNES tertiary EFL writing teachers’ actual practices in regard to written feedback provision. The results of our study showed that the two groups of teachers displayed similar feedback practices in scope and strategies. In terms of feedback scope, both utilized a comprehensive approach to provide feedback, but NNES teachers gave significantly more feedback points than NES teachers. As for feedback strategies, the two groups of teachers combined direct and indirect feedback strategies. However, their feedback practices differed greatly with regard to focus: NES teachers put more emphasis on global issues while NNES teachers gave priority to local issues while offering written feedback.

The findings of this study extend the current literature on how L2 writing teachers implement written feedback in their specific pedagogical contexts. The previous relevant studies examine this issue without taking into account teachers’ sociocultural/language backgrounds. However, explicitly setting teachers’ L1 as a variable, this study compares NES and NNES teachers’ written feedback practices, which enables us to have a better understanding of such an issue.

Also, this study yields useful pedagogical implications. Firstly, several “best practices” of feedback provision have been proposed (Ferris, 2014; Lee, 2019). However, they were not fully translated into NES and NNES teachers’ actual written feedback practices in mainland China. As such, teacher training programs in relation to written feedback provision should be encouraged. L2 writing teachers should be provided access to different forms of training sessions such as workshops, seminars, and short-term courses. With these sessions, NES and NNES teachers are probably empowered to reflect on their own feedback practices, acquire cutting-edge pedagogical knowledge about written feedback, and maximize the efficacy of their written feedback. Secondly, it is a long-term enterprise for both NES and NNES teachers to foster and develop their feedback literacy. Thus, they should become life-long learners and proactively engage themselves in professional learning. Aside from teacher education programs, they can benefit from self-training. NES and NNES teachers can train themselves to optimize their written feedback practices. Specifically, front-line L2 writing teachers can peruse the state-of-the-art research articles, attend domestic and/or international academic conferences, and communicate their feedback practices with experienced teachers or teacher educators. Such different types of self-training would help NES and NNES teachers tackle the difficulties and challenges when they implement written feedback, and improve their expertise in feedback provision. Finally, our study revealed that NES and NNES teachers had divergent orientations in focus while providing feedback (global feedback vs. local feedback). In this sense, the two groups of teachers should learn from each other and observe each other’s written feedback on students’ writing to understand how to provide feedback on global issues or local issues. Also, a co-feedback model can probably be tapped by Chinese universities and colleges. Specifically, NES teachers, particularly those with EFL writing teaching experience can be invited to cooperate with NNES teachers: NES teachers provide global feedback, while their NNES colleagues offer local feedback when responding to a piece of writing. Such a model enables NES and NNES teachers to complement each other and exercise their respective advantages in L2 writing assessment. As such, L2 learners may achieve a balanced development in both the local and global dimensions of writing.

Not surprisingly, this study is not free from limitations. Firstly, as one of the first attempts to compare NES and NNES teachers’ feedback practices, this exploratory study only included eight teachers. Future studies should enlarge the sample size so that an understanding of this issue can be broadened. Additionally, this study investigates NES and NNES teachers’ written feedback practices at only one point in time instead of over time. Given that teachers’ instructional practices are not static but dynamic, longitudinal studies are needed to investigate this issue. Finally, due to the research focus, this study primarily collected data from teachers’ feedback on their students’ writing samples. Future studies can employ other instruments including semi-structured interviews, stimulated recalls, and think-aloud to collect data. By doing so, the issues such as teachers’ decision-making about feedback provision in relation to their demographic backgrounds and the factors contributing to the differences of NES and NNES teachers’ feedback practices can be examined, which can provide more insights into how the two groups of teachers implement written feedback.

## Data Availability Statement

The raw data supporting the conclusions of this article will be made available by the authors, without undue reservation, to any qualified researcher.

## Ethics Statement

The studies involving human participants were reviewed and approved by the University of Auckland Human Ethics Committee. The patients/participants provided their written informed consent to participate in this study.

## Author Contributions

XC conceived of the initial idea, fine-tuned by LZ. XC designed the study, collected and analyzed the data, and drafted the manuscript. LZ revised, proofread, and finalized the manuscript for submission as the corresponding author. Both authors contributed to the article and approved the submitted version.

## Conflict of Interest

The authors declare that the research was conducted in the absence of any commercial or financial relationships that could be construed as a potential conflict of interest.

## Footnotes

1 TEM-4 (Test for English Majors Band 4) is a large-scale and well-established test in mainland China, aiming to evaluate English major students’ English proficiency.

2 Other 10 feedback points were positive comments provided by NES teachers.
